# Automated Detection of Conformational Epitopes Using Phage Display Peptide Sequences

**DOI:** 10.4137/bbi.s2745

**Published:** 2009-07-01

**Authors:** Surendra S Negi, Werner Braun

**Affiliations:** Sealy Center for Structural Biology and Molecular Biophysics, Department of Biochemistry and Molecular Biology, University of Texas Medical Branch, Galveston TX, 77555-0857, USA. Email: webraun@utmb.edu

**Keywords:** conformational epitopes, peptides, phage display

## Abstract

**Background:**

Precise determination of conformational epitopes of neutralizing antibodies represents a key step in the rational design of novel vaccines. A powerful experimental method to gain insights on the physical chemical nature of conformational epitopes is the selection of linear peptides that bind with high affinities to a monoclonal antibody of interest by phage display technology. However, the structural characterization of conformational epitopes from these mimotopes is not straightforward, and in the past the interpretation of peptide sequences from phage display experiments focused on linear sequence analysis to find a consensus sequence or common sequence motifs.

**Results:**

We present a fully automated search method, EpiSearch that predicts the possible location of conformational epitopes on the surface of an antigen. The algorithm uses peptide sequences from phage display experiments as input, and ranks all surface exposed patches according to the frequency distribution of similar residues in the peptides and in the patch. We have tested the performance of the EpiSearch algorithm for six experimental data sets of phage display experiments, the human epidermal growth factor receptor-2 (HER-2/neu), the antibody mAb Bo2C11 targeting the C_2_ domain of FVIII, antibodies mAb 17b and mAb b12 of the HIV envelope protein gp120, mAb 13b5 targeting HIV-1 capsid protein and 80R of the SARS coronavirus spike protein. In all these examples the conformational epitopes as determined by the X-ray crystal structures of the antibody-antigen complexes, were found within the highest scoring patches of EpiSearch, covering in most cases more than 50% residues of experimental observed conformational epitopes. Input options of the program include mapping of a single peptide or a set of peptides on the antigen structure, and the results of the calculation can be visualized on our interactive web server.

**Availability:**

Users can access the EpiSearch from our web server http://curie.utmb.edu/episearch.html

## Introduction

The identification of conformational epitopes in antibody-antigen interaction is a crucial step for the rational design of novel drugs and vaccines. The most direct experimental method to accurately define an epitope is to determine the 3D structure of the antibody-antigen complex by X-ray crystallography^1^1–5 however these methods are time-consuming and need purified protein complexes. An alternative approach makes use of screening biological peptide libraries with antibodies to identify peptides that mimic conformational epitopes. 6–8 A library of peptides is generated by filamentous phages that display a random set of peptides on their surfaces. The antigen binding site on the antibody, used in the screening, will select those peptides from the random phase libraries that have a subset of similar residues in common with the conformational epitope of the antigen. In iterative procedures of biopanning the libraries with the antibody of interest, residues important for binding can then be enriched in the phage sequences. Phage display methods have been shown to experimentally identify residues critical for antigen-antibody interactions. 6–14 It has been shown that the peptides obtained from random phage libraries can mimic conformational epitopes, thus they are also referred as mimotopes. 9,13–17 The peptide sequences of mimotopes are found quite often be different from those of the natural antigen, thus the assignment of conformational epitopes from the peptide sequences can not be done by sequence alignment of the selected peptides with the sequence of the antigen. Conformational epitopes are formed in most cases by a small group of amino acid residues scattered along the protein sequence yet form contiguous clusters of residues on the protein surface.^9^,^17^,^18^ Methods using only the linear sequence of the antigen are thus not applicable in most cases.

In the current work, we developed a fully automated computational method, EpiSearch (http://curie.utmb.edu/episearch.html) to map a conformational epitope on the antigen protein surface from phage display sequences without manual intervention. EpiSearch is based on a patch analysis that identifies spatial contiguous clusters of residues on the surface of the antigen with similar physical-chemical properties as found in the phage display sequences. Similarity of residues is measured by a physical-chemical property distance that was derived from five descriptors of amino acid residues.^19^–^24^ The performance of the EpiSearch algorithm has been tested for six experimental data sets of phage display experiments and compared to the conformational epitopes as found by the X-ray crystal structures of the corresponding antibody-antigen complexes. The highest-scoring patch of EpiSearch was found in all the test cases overlapping with the experimentally observed conformational epitopes, and the predicted conformational epitopes cover in most cases more than 50% residues of the conformational epitopes. In the online version of the EpiSearch method, users can map a single consensus sequence or up to thirty peptide sequences at a time. The output results from EpiSearch are displayed in a table and automatically mapped on the 3D structure using Jmol molecule viewer.^25^

## Methods

**Figure 1.**
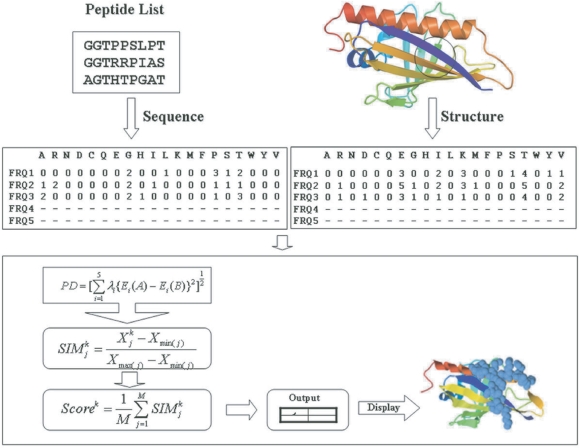
Flow chart of the EpiSearch method used to map peptide sequences obtained from phage display experiment onto 3D protein structure.

The conformational epitopes on the protein surface are predicted by an automated sequence analysis of all phage display sequences and a comparison to the distributions of amino acids on three-dimensional patches on the protein surface. The amino acid compositions of the linear and 3D profiles are compared and quantified in a score function for each patch on the protein surface. The highest scoring patches are listed in the output files and are also displayed on the surface of the protein. An overview of the implemented procedure in EpiSearch is given in Figure 1.

### Amino acid composition in the phage display sequences

The input of EpiSearch uses the set of *M* sequences from phage display experiments (labeled *j* = 1, ..., *M*) that mimic the surface residues of an antibody binding sites and the 3D structure of the antigen. The frequency distributions of residues for these peptide sequences are stored in a matrix *mat1* (*M*, 20) with *M* rows and 20 columns.

### Amino acid composition of the surface patches

The protein surface of the antigen is decomposed in overlapping surface patches around each solvent accessible amino acid residue. Therefore, in a protein with *N* solvent accessible residues, we have *N* number of surface patches, labeled *k* = 1, ..., *N*. The solvent accessible surface area of each residue is calculated by GetArea^26^ with a probe radius of 1.4Å. A residue is considered to be solvent exposed if its solvent accessible surface area is greater than 10Å^2^ otherwise the residues are considered as buried in the protein.^22^,^24^ The surface exposed residues are represented by their *Cβ* atom (*Cα* atom in case of *Gly* residue). Using the *Cβ* position of each residue (*Cα* atom in case of *Gly* residue), a surface patch of radius *R* is drawn around each surface exposed residues. The frequency distribution of amino acid residues in each patch is calculated and saved into a second matrix *mat2* (*N*, 20). A patch size of radius 12Å was used for all calculations with EpiSearch. Empirically, a patch size of radius 12Å was chosen to cover the surface area of all the residues present in the input peptide sequences (in most of the test cases the input peptide sequence is 12 residues long). An increase of the patch size may increase the sensitivity of the method but at the same time it will also decrease the precision of the method. On other hand a decrease in patch size may predict a smaller number of residues and therefore decrease the sensitivity of the method.

### Criterion for matching residues

In order to map a peptide on the protein surface, we calculate the property distance *PD* (*A*, *B*) of residues *A* in the peptide sequences and residues *B* in each patch as:

(1) $$PD(A,B)=\sqrt{\sum_{i}\lambda_{i}{\left(E_{i}(A)-E_{i}(B)\right)}^{2}}$$

where the *E_i_* (*i* = 1,5) are five quantitative descriptors representing physicochemical properties of amino acids and *λ_i_* are the eigen values of the *i*th component of *E*.^19^,^21^ If two amino acid residues are identical, their *PD* value is zero, and similar amino acid residues have small *PD* values. We used a cutoff value of *PD* = 8.0 as the criterion to have matching residues between phage display sequences and surface patches. We found empirically that this *PD* value represents a good threshold for residues with similar properties. The number of matching residues in each peptide *j* and patch k are then stored in a new matrix $X_{j}^{k}$.

### Score function of surface patches

Finally, for a given peptide *j*, the total number of predicted residues in each patch *k* is normalized using

(2) $$SIM_{j}^{k}=\frac{X_{j}^{k}-X_{{\text{min}}_{j}}}{X_{{\text{max}}_{j}}-X_{{\text{min}}_{j}}}\;\;\text{for}\;\;k=1,2,\ldots ,n$$

where, *X*_min_*_j_* and *X*_max_*_j_* are the minimum and maximum number of matching residues present in all patches for a given peptide sequence *j*. This normalization transforms the number of matching residues for each peptide *j* to a uniform range; i.e. a $SIM_{j}^{k}$ value of 1 represent a patch k with the maximum number of matching residues for a given peptide *j*, and a $SIM_{j}^{k}$ value of 0 represent a patch *k* with the minimum number of matches. The process is repeated over all input peptide sequences *j*. We then select only patches as potential candidates for conformational epitopes if they have $SIM_{j}^{k}$ values greater than 0.5 for all peptides *j*; i.e. patches have a reasonable good match with all peptides. The final score, Score*^k^*, of each selected patch *k* is then calculated as the average over all *M* peptides,

(3) $${\text{Score}}^{k}=\frac{1}{M}\sum_{j=1}^{M}SIM_{j}^{k}$$

The highest scoring patch is selected as the predicted patch, and those individual residues within the predicted patch that match at least one of the peptide sequences are predicted as part of the conformational epitope.

### Definition of the observed conformational epitope derived from the X-ray structure of the antibody-antigen complex

A residue is considered as part of the observed epitope in the X-ray crystal-structure of the antibody-antigen complex, if the residue changes its solvent accessible surface area by more than 10Å^2^ in complex formation.

## Results

### Mapping of trastuzumab peptides

As a first test for the performance of our procedure we selected the trastuzumab epitope. Trastuzumab, also known under the trade name Herceptin, is a humanized monoclonal antibody that inhibits growth of Her-2/neu expressing tumor cells, and has been approved as immunotherapy for breast cancer patients.^27^–^30^ The group of E. Jensen-Jarolim has isolated five peptides each 12 residue long from phage display experiments to characterize the epitope of the antibody trastuzumab on the human epidermal growth factor receptor-2, Her-2/neu.^28^,^29^ The conformational epitope of trastuzumab on Her-2/neu has also been determined by a co-crystal structure of a trastuzumab Fab fragment with the extra cellular domain of the HER2 receptor, PDB id: 1N8Z.^30^ Main residues representing the conformational trastuzumab epitope are located in the C terminal of domain-IV with the three segments of amino acids 557–561, 569–573 and 591–603 respectively. Multiple sequence alignment shows no consensus region between the sequences of the phage display peptides and the HER-2/neu sequence.^28^

**Figure 2.**
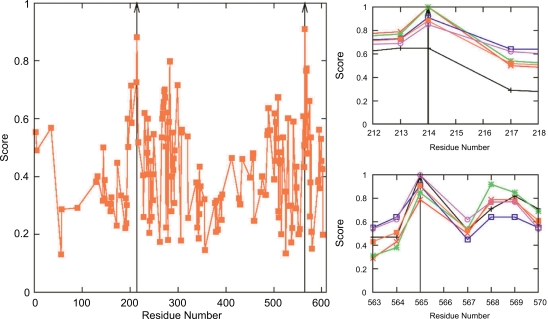
**A**) Average score profile of five trastuzumab peptides CQMWAPQWGPDC, CKLYWADGELTC, CKLYWADGEFTC, CVDYHYEGTITC, and CVDYHYEGAITC in each patch centered on the surface exposed residues of HER2 protein surface is shown. **B–C**) shows the score profile for the five peptides at the two high scoring patches centered at residues 214 and 565, respectively.

High scoring patches on Her-2/neu were predicted by EpiSearch in two locations ranging from residues 200 to 300 and 550 to 600 (Fig. 2A). The patch with the highest score (0.910) was found with the center at residue C565, and the second highest score was found centered at residue C214 (Score: 0.882). The individual score profiles for all 5 peptides are shown in a close-up views in Figure 2B and Figure 2C. Patches at the third and fourth rank were found at K569 with a score 0.774 and at Y568 (Score: 0.766) overlapping with the patch at C565. Other high scoring patches at residues 272 and 283 are near the patch centered at C214 on the surface of Her-2/neu.

**Figure 3.**
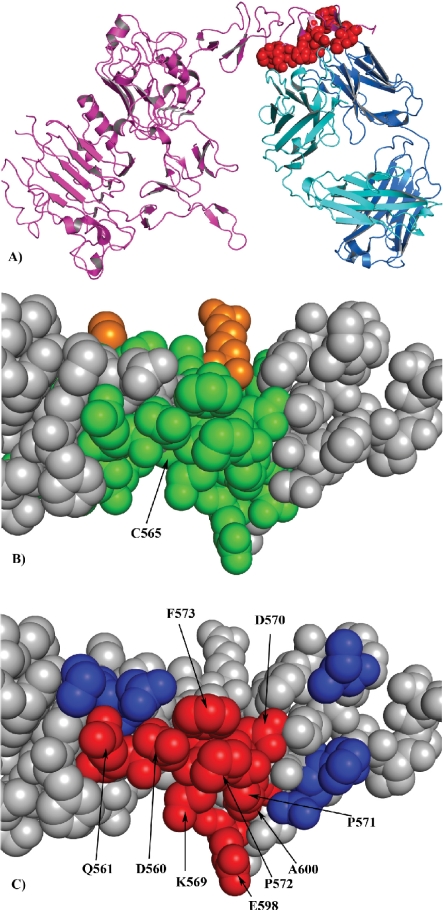
Comparison of the genuine epitope site on HER2 surface obtained from X-ray crystallography with the epitope site predicted by the EpiSearch method. **A**) The amino acids at the genuine epitope site on HER-2 surface (red). **B**) The amino acids predicted by the EpiSearch method present in the highest scoring patch centered on C565 and also present in the input peptide sequences (green), while the residues not present in the input peptide sequences are shown in orange. **C**) Amino acids correctly predicted and present in the genuine epitope binding site on HER2 surface (red), while the residues present in the epitope site and not predicted are shown in blue.

The patch at residue C565 with the highest score coincides to a large extent with the experimentally determined epitope of the antibody—Her-2/neu complex which is shown in red in Figure 3A. A detailed view of the predicted patch site at C565 shows residues on the surface of Her-2/neu that match residues in the peptide sequences, i.e. these are the predicted residues within the patch (in green) and other residues of the patch are shown in orange (Fig. 3B). Most of these predicted residues are correct, as shown in red in Figure 3C, and only a few residues of the experimentally determined epitope are not predicted (shown in blue). The second highest scoring patch at residue C214 was located on the other face of the HER-2, and is not part of the interface of the antibody—Her-2/neu complex. However both the patches share a large fraction of amino acids present in the input peptide sequences. This patch is also predicted as one of the top rank cluster in Mapitope analysis.^17^

### Mapping of mAb Bo2C11 epitopes

**Figure 4.**
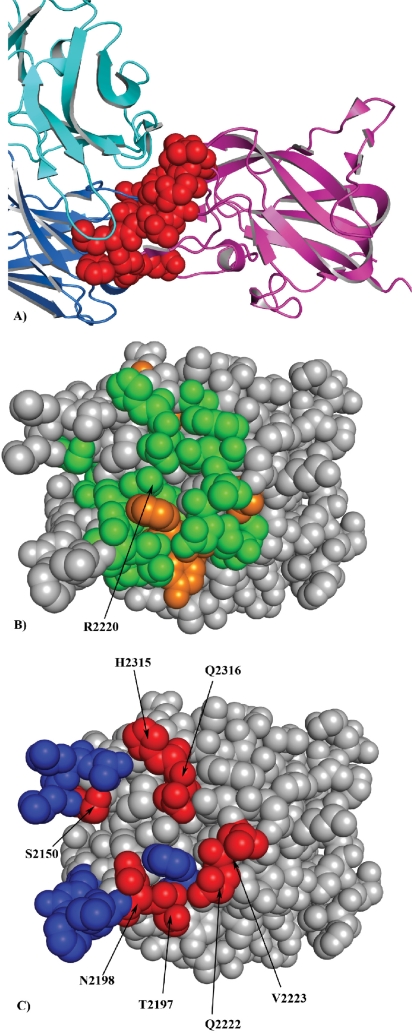
**A**) Amino acids in C_2_ domain of FVIII (red) complex with BO2C11 Fab. **B**) The residues predicted by the EpiSearch method in the highest scoring patch and present in the input peptide sequences are shown in green while the residues not present in the input peptide sequences are shown in orange. **C**) Amino acids correctly predicted and present in the BO2C11 binding site (red), while the residues present in the BO2C11 binding site and not predicted are shown in blue.

Next, we compared the performance of the EpiSearch method to map the epitopes recognized by mAb Bo2C11 on the C_2_ domain of coagulation factor VIII (FVIII). Bo2C11 is a human anti FVIII monoclonal antibody that binds to C_2_ domain of FVIII, an essential cofactor in the intrinsic pathway of blood coagulation. Deficiency of FVIII results in bleeding disorder commonly known as hemophilia A. 9,31–33 We used a list of 27 peptides each 12 residue long selected against mAb Bo2C11 targeting the C_2_ domain of FVIII.^31^ Using the X-ray crystal structure of the C_2_ domain of FVIII^34^ (PDB id:1IQD), EpiSearch method predicted a potential epitope on the C_2_ domain of FVIII that correspond to a patch centered at the residue R2220 with the highest score (0.937). The predicted residues form a large part of the conformational epitope as determined by the X-ray crystal structure of the Bo2C11 mAb-C_2_ domain (Fig. 4A). The residue R2220 is completely buried at the Fab interface and forms a salt bridge with the residue D102 on the Fab Bo2C11 while residues H2315 and Q2316 play an important role in polar interactions at the antibody interface.^34^ Other three patches predicted by EpiSearch method are centered on the residues P2205 (Score: 0.881), E2228 (Score: 0.804) and Y2195 (Score: 0.769) respectively. The residues predicted in these patches are also present in the Bo2C11 binding site. The predicted residues from the highest scoring patch are shown in Figure 4B, and to a large extent correctly predicted to be part of the interface are shown in Figure 4C.

### Mapping of mAb b12 and mAb 17b epitopes

In order to map the conformational epitopes of mAb b12^13^ onto the crystal structure of gp120 (PDB id: 2NY7) we selected nineteen 12-mer cysteine looped peptides. Using default value of parameters, EpiSearch method predicted a high scoring patch centered on residue T283 (Score: 0.943). A comparison between the residues located in the predicted patch with those located at the interface of gp120-mAb b12 complex revealed that the EpiSearch method correctly predicted the residues N280, A281, K282, R469, T455, G472, G473 and M475 as part of the conformational epitope. We also observed that the residues D279, N280, A281, K282 and T283 predicted in the high scoring patch coincide with the CD4 binding site in the gp120.^13^,^35^ When the analysis was repeated after removing the terminal cysteine residues from the peptide sequences, a high scoring patch centered at T283 (Score: 0.911) was predicted. This indicates that the method is able to predict the location of the conformational epitope correctly with or without the terminal cysteine residue.

We also mapped the conformational epitopes of mAb 17b onto the crystal structure of gp120^36^ using eleven 12-mer cysteine looped peptides of the mAb 17b.^18^ The EpiSearch analysis predicted two high scoring patches centered on residues L116 (Score: 0.780) and L122 (Score: 0.683). The patches partially overlap with each other and share more than 70% of the residues present in the input peptide sequences. We observed that the accuracy of the method was lowered by removing the terminal cysteine residues from the peptide sequences. This may be attributed to the presence of cysteine residue in the epitope binding site which has been shown critical for stabilizing the gp120 core. 35–37

### Mapping of mAb 13b5 epitopes

A list of fourteen 12-mer cysteine-loop peptides of mAb 13b5^18^ that bind HIV-1 capsid protein (p24)^38^,^39^ (PDB id: 1E6J) were selected. Using default value of the parameters, EpiSearch method predicted two top scoring patches centered on residues M214 (Score: 0.813) and R100 (Score: 0.805). The patch M214 was located in the C-terminal while the patch centered at R100 was located in the N-terminal region of p24. Earlier, the crystal structure of HIV-1 p24-mAb 13b5 shows that the antibody recognizes a region in the C-terminal domain of p24 between the residues L205 to E213.^39^ However, the dimeric structure of the p24, as observed in the type II crystal, shows a dimer interface between N-terminal domain of one molecule with the C-terminal domain of two fold related molecule.^38^ This observation brings the locations of both predicted patches in a close proximity in the dimer structure. Both high scoring patches centered at M214 and R100 were also obtained when the analysis was repeated after removal of the terminal cysteine residues from the input sequences. The location of the predicted patch M214 on HIV-1 p24 surface corresponds to the experimentally observed conformational epitope.

### Mapping neutralizing epitope 80R on SARS CoV spike protein

In this example, we selected a list of eighteen peptide sequences each fifteen residues long derived from the libraries of random linear peptides^40^ and mapped them onto the surface of SARS Coronavirus Spike protein receptor binding domain 2 (PDB id: 2AJF).^41^ Using the default value of input parameters, we obtained two high scoring patches centered at residues F460 (Score: 0.844) and L443 (Score: 0.804). The amino acids predicted by the EpiSearch method were observed at the interface site of the spike protein and its receptor ACE2, and may represent the possible binding site for anti-SARS monoclonal antibody, 80R. When the peptide sequences were tested independently, we found that with the exception of the peptide no 12 and 16, ten peptides mapped correctly in the interface region while five mapped in a patch centered at residue L355, a site also predicted by Tarnovitski et al^40^ on SARS Coronoavirus. Very low sequence identity among the input peptide sequences may be responsible for the prediction of multiple binding sites. However, all the peptide sequences satisfying the criteria defined by equation (3) were found in a patch centered at residue F460.

## Performance of the Method

The EpiSearch method was successfully validated using six independent test cases where input peptide sequences from phage display experiments as well as the X-ray co-crystal structures of antigen-antibody were available. Table 1 gives an overview of the accuracy of EpiSearch method and shows that the method correctly predicted the location of conformational epitopes. Despite the low sequence similarity among the input peptide sequences, the residues in the highest scoring patch shared approximately 70% sequence identity with the residues in the input peptide sequences. In our analysis, we observed that, though all the residues in the input peptide sequences were not present in the epitope binding sites, a highest scoring patch correctly predicted the location of the conformational epitopes as found in the crystal structure. Except in case of mAb 17b peptides, the extent of coverage to locate the conformational epitope site in all the test cases was found to be more than 50% (Table 1). In case of mAb 17b the second high scoring patch performed better than the top scoring patch. The second high scoring patch partially overlapped with the top scoring patch and contained seven of the eleven residues present in the mAb 17b interface. In case of Trastuzumab, mAb 17b, mAb b12 and mAb 13b5 peptides, we also determined the influence of terminal cysteine (Cys) residues on the quality of the results. Experimentally, the terminal cysteine residues were introduced to constrain the peptides to a cyclic structure and might influence the selection process. We observed that in most of the cases the result was not sensitive to the presence of the Cys as terminal residues. However, in the case of mAb 17b bound gp120 the constrained loop peptides gave a better result than the analysis performed without the terminal Cys residues, which is evident from the presence of a Cys residue in the epitope binding site of gp120.^35^,^36^

The performance of the EpiSearch method was compared with the online version of the PepSurf method (http://pepitope.tau.ac.il/),^42^ see Table 2. We observed that PepSurf method groups the input peptide sequences based on their sequence identity and maps the high scoring group on to antigen surface. However in case of the EpiSearch method all input peptide sequences satisfying equation 2 were mapped on the antigen surface.

## Discussion and Conclusions

It is generally believed that not all amino acids in a protein interface contribute to the affinity of binding of a protein complex in a uniform way, and major contributions to the binding energies of a protein complex are composed from a small fraction of amino acids present in the protein surface, known as hot spots.^22^,^43^–^47^ However, flexible peptides that bind to the antigen binding site of an antibody can use different binding modes not necessarily identical to a relatively rigid protein surface. The EpiSearch method assumes that all peptides that are selected during the bio-panning process use a subset of the actual antigen-antibody interactions, and that the ensemble of all sequences sample most of favourable interactions in the protein-antibody interface. Therefore only surface patches that have a relative high number of matching residues for all peptide sequences are selected in our prediction process. We also assume that some of the physical pair interactions in the protein-antibody interface can be replaced by residue pairs with similar physicochemical properties, e.g. hydrophobic interactions, polar-polar or charge-charge interactions. Thus our method does not only match residues of identical types, but also residues with similar physicochemical properties, i.e. with low PD values.

The number of antigen-antibody complexes that are experimentally determined by X-ray crystallography is continuously increasing in the protein data bank,^48^ however mapping the conformational epitopes by phage display experiments is an alternative method to the 3D-structure determination in case a high affinity complex can not be isolated. The phage display technique first introduced by G. Smith^49^ has been successfully used to screen a large peptide libraries that binds to a specific antibody. Simple comparison of the linear sequences of the selected peptides to the sequence of the antigen is in most cases not possible, as the peptides selected from the phage display experiment represent discontinuous fragments of amino acids that may not have a high sequence homology to the antigen. The test cases we present here show that our EpiSearch method is a reliable technique to help in the interpretation of the peptide sequences.

A number of computational methods used to identify the epitopes on the antigen structure have been reported earlier.^9^,^13^,^15^–^18^,^33^,^37^,^42^,^50^–^52^ Most of these methods are either computationally expensive or unable to process a large number of peptide sequences at a time as discussed in detail by Mayrose et al.^37^ EnShell-Seijffers et al^18^ described a method that can be used to find the most frequent tandem pairs of amino acids selected from the phage display peptide library. The algorithm defines the statistically significant pairs of amino acids (AAP) by calculating the distance between two *Cα* atoms of adjacent residues. The AAP can be considered if the distance between to consecutive *Cα* is less than a given threshold value, *D* (the distance between alpha carbon of any given pair). Each peptide sequence obtained from the phage libraries are deconvoluted into AAPs and the most significant pairs were mapped on the protein surface. A new version of Enshell et al algorithm known as Mapitope,^17^ is available which depends on the selection of the distance between *Cα* atoms of any given pair and the statistical threshold values. Other methods, Findmap,^53^ 3DEX^16^ and MIMOX^33^ uses one peptide sequence (or a consensus peptide sequence) at a time while PepSurf 37,42 becomes highly CPU time intensive when longer peptides are used^37^ and the performance of the MIMOP^9^ relies on the multiple sequence alignment of peptide sequences. The ability of the EpiSearch method to predict genuine epitope binding site on the antigen surface was also tested using a single peptide sequence as well as a group of three, five and if possible nine, fourteen and twenty sequences. As shown in Table 1, the EpiSearch method correctly mapped the location of conformation epitopes. However, subtle differences were observed when the input peptide sequences shared a low sequence identity.^16^,^17^,^33^,^42^

Major advantages of EpiSearch are its flexibility for handling a single peptide sequence or a group of peptide sequences and its efficiency to process a large number of peptide sequences in a very short time. Based on tested examples shown here the average processing time is about one minute which may vary depending on the size of the input protein data file used. Also the EpiSearch method is flexible enough and allows the user to vary the cut off value for solvent accessible surface area of amino acids and the patch size. For example, in the case of Trastuzumab peptides, the method correctly predicts six residues using a 10Å patch size and three residues using a patch size of 8Å compared to nine residues using a patch size of 12Å. Similarly, in the case of mAb Bo2C11, the method correctly predicts five residues using a patch size of 10Å compared to seven residues using a patch size of 12Å. A further decrease in patch size predicts only four residues. A patch size below 10Å can accommodate fewer residues present in the 12-mer peptide and therefore recommends to use peptide sequences up to seven (7-mer) or nine (9-mer) residues long. The results obtained from the web server are shown interactively in a tabular form as well on the protein surface using JMOL molecular viewer.^25^ In addition, using the information about the surface exposed residues, EpiSearch method can be used to design peptides that might interfere with protein-protein interactions. Also, we hope that the results obtained from the EpiSearch method in combination with the information available from site directed mutagenesis will provide a vital resource to understand the nature of the antigen-antibody interaction in greater details. An automated version of the method is freely available online at http://curie.utmb.edu/episearch.html.

## Figures and Tables

**Table 1.** Performance of the EpiSearch method for six independent test cases. Results are shown for the highest scoring patch, for the gp120-mAb 17b complex we also include the data for the second scoring patch. In all six cases the highest scoring patches overlap with the experimentally known epitopes as shown in the number of correctly predicted residues (column 6). This number is then compared to the number of all residues in the epitope sites (column 4), given as coverage, TP/(TP = FN), in column 7, and also compared to the number of all predicted residues in that patch, given as overlap ratio, TP/(TP = FP), in column 8. TP = correctly predicted amino acids, FN = amino acids not predicted and FP = amino acids predicted incorrectly on the protein surface.

**Name of antibody**	**Number of input peptides**	**Highest patch score**	Number of interface residues*	**Number of residues in a patch**	**Number of correctly predicted residues**	**Extent of coverage**	**Overlap ratio**	**CPU Time (second)**
Trastuzumab	5	0.91	14	27	9	0.64	0.33	62
mAb Bo2C11	27	0.93	14	20	7	0.50	0.35	43
mAb b12	19	0.94	18	26	8	0.44	0.31	46
mAb 17b^†1^	11	0.78	11	27	2	0.18	0.07	29
mAb 17b^†2^	11	0.68	11	19	7	0.64	0.37	29
mAb 13b5	14	0.81	12	21	12	1.00	0.57	21
80R	18	0.84	16	22	6	0.38	0.27	24

† Both first^1^ and second^2^ high scoring patches are shown.

* Interface residues are calculated if the change is ASA more than 10Å^2^ in complex formation.

**Table 2.** Performance of the PepSurf method using five independent test cases is shown. In case of trastuzumab epitope a third high scoring cluster on Her2 surface in contact with the trastuzumab was selected.

**Name of antibody**	**Number of input peptides**	**Highest Patch score**	Number of interface residues*	**Number of residues in a patch**	**Number of correctly predicted residues**	**Extent of coverage#**	**Overlap ratio^1^**	**CPU Time (second)**
Trastuzumab†	5	10.567	14	11	4	0.29	0.36	–
mAb Bo2C11	27	771.98	14	22	2	0.14	0.09	–
mAb b12	19	745.45	18	39	3	0.16	0.10	–
mAb 17b	11	306.38	11	29	6	0.55	0.20	–
mAb 13b5	14	635.15	12	26	11	0.92	0.42	–
80R$	–	–	–	–	–	–	–	–

† Result from correctly predicted cluster (third) is shown.

# Relative to actually observed in epitope TP/(TP = FN) (extent of coverage),

1 Relative to total predicted residues TP/(TP = FP) (overall ratio) as defined in table 1.

$ No results available from PepSurf web server as PepSurf can not accept peptide sequences longer than 14 amino acids.
